# Cases of Sciatica Cured with Strychnine

**Published:** 1858

**Authors:** O. C. Gibbs

**Affiliations:** Frewsburg, Chatauque Co., N. Y.


### A Case of Sciatica Cured with Strychnia.

BY O. C. GIBBS, M. D., FREWSBURG, CHATAUQUE CO., N. Y.

The disease usually denominated Sciatica, is often an extremely painful affection, and, what is worse, is notoriously obstinate of cure. The case we are about to relate, is interesting from the promptness with which it yielded to strychnine, after a failure of a great variety of other remedies.

March 6th, 1857, we were called to see Mr. H., aged about 45 years. He was laboring under extreme pain in one hip and leg. The pain was particularly severe between the great trochanter and the ischium, extending thence downward upon the outside and back of the thigh to about half way between the knee and ankle, where the pain was nearly as severe as in the hip. There was no swelling or redness of the affected parts.—The patient was confined to the bed and could lie only upon the well side. The only relief he could get from this position was by turning upon his face and standing upon his hands and the knee of the well leg. With the exception of a slight billious tinge to the complexion and a little acceleration of the pulse, the general health was not materially affected. Slight pain and tenderness were constant, but severe pain occurred only in paroxysms.

The patient stated that he had a similar attack about three years since, which proved extremely obstinate, resisting all treatment for a year-and-a-half, but finally passed gradually away while taking the tincture of colchicum and the ammoniated tincture of gum guaiac. At that time he was in the care of a very intelligent and skilful physician, my friend Dr. Axtell, now of Jamestown, N. Y.

He had been suffering from the present attack about two weeks, during the most of which time he had taken regularly the tincture of cholchicum and the ammoniated tincture of gum guaiac. Looking upon the case as more neuralgic than rheumatic, we gave him a cathartic containing a small portion of calomel, and ordered it to be repeated every second day. We also ordered two grains of sulphate of quinine and one-fourth of a grain of sulphate of morphine, to be given every six hours. This prescription was continued for a week without benefit. We now ordered the subcarbonate of iron in half drachm doses, extract belladonna in half grain doses, and oil turpentine in doses of fifteen drops, each to be repeated every six hours. This prescription was continued for a week, also, without benefit. We now ordered the tincture of cimicifuga in drachm doses, the iodide of potassium in two grain doses, and the oil of turpentine was continued as before, each every six hours. This treatment was continued also for a week, apparently without benefit. We now ordered small doses of calomel with Dover's powders, also tincture of colchicum in appropriate doses; we also ordered blisters over the great trochanter, and at the seat of pain at the middle of the leg, the cuticle to be removed, after blistering, and sprinkled with morphine. It may not be amiss to state here, that we had not previously blistered because, in the previous attack, he had been blistered and cupped repeatedly without benefit. The calomel was continued until the system was slightly under its influence, when it was discontinued and the iodide of potassium substituted. This plan of treatment was continued for a week or ten days, without any improvement; in fact the patient seemed worse in general health, and the paroxysms of pain were more severe than when treatment was commenced. The patient became impatient and restless under treatment, and, for some time, had been in the habit of using opium at his or his wife's discretion, as the only means of procuring relief from pain.

We now took two grains of strychnia in crystals and put it with two ounces of water, slightly acidulating the water with sulphuric acid. We also took four grains of podophylline and five of sulphate of morphine, rubbing them up with sugar and dividing into twenty powders. We ordered thirty drops of the solution of strychnia, also one of the powders, to be taken each three times a day. The patient had no severe paroxysms of pain after this, and within three weeks from the time of commencing the strychnia he went down the Alleghany and Ohio rivers as a pilot on a lumber raft, and up to the present time he remains free from a return of the affection.

We have no recollection of ever having seen strychnia recommended in this form of disease. The remedy was held under consideration quite early in the treatment, but failing to find authority for such administration, its use was postponed until repeated failures seemed to justify a trial of any remedy that theoretically promised benefit. It is, of course, uncertain whether the remedy in this case stands in relation to the cure as cause to effect. Subsequent observations can only establish this; for one case, we are well aware, is nearly valueless in establishing the remedial powers of any article over any disease. —*American Medical* (N. Y.) *Monthly for Sept.*

